# Wild-Type *Drosophila melanogaster* Strains Respond Differentially to Rotenone Exposure

**DOI:** 10.17912/micropub.biology.001380

**Published:** 2025-01-27

**Authors:** Shiva Chaudhary, Shreyas Mohan Iyer, Meghana Tare

**Affiliations:** 1 Department of Biological Sciences, Birla Institute of Technology and Science, Pilani, Pilāni, Rajasthan, India

## Abstract

*Drosophila melanogaster* has been established as a reliable *in vivo* model for studying human diseases. However, the varied designs of such studies and the different origins of the strains have significantly contributed to metabolic and molecular differences between strains. Parkinson’s disease (PD) is a neurodegenerative disorder involving the loss of dopaminergic neurons, leading to various motor and non-motor symptoms including but not limited to bradykinesia, postural instability, cognitive decline, and gut dysbiosis. Chronic exposure to toxins such as rotenone can induce neuronal cell death. We have developed a sporadic PD model by direct feeding of rotenone-supplemented food to *Drosophila melanogaster* wild-type strains, which has previously been shown to cause neuronal cell death and used to mimic PD in *Drosophila.* Upon exposure to rotenone in two wild-type strains ( *Oregon-R* and *Canton-S)* , differences in their climbing ability and lifespan were monitored. We found that the degree of motor defects upon rotenone exposure is higher in *Oregon-R* compared to age-matched *Canton-S* flies. We also observed that the *Canton-S* flies (rotenone-fed and non-rotenone-fed) exhibited a lower survival percentage than *Oregon-R* flies. However, the climbing defects in *Canton-S* flies are not as pronounced as in *Oregon-R* flies. The mechanism(s) involved in such differential effects in different wild-type *Drosophila* strains are yet to be explored and may provide a perspective on differential symptoms of PD patients belonging to different demographics.

**Figure 1. Differences in Wild Type strains of flies in climbing abilities and survival of flies:**
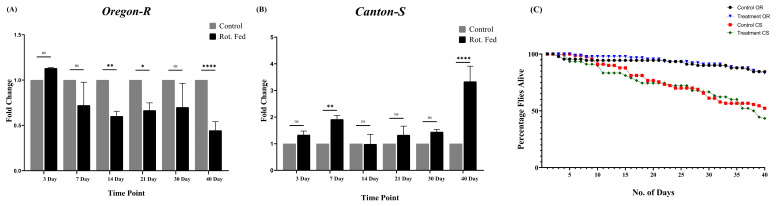
Negative geotaxis assay or climbing assay of *Oregon-R* and *Canton-S* flies depicting changes in locomotor activity with time (A–B). The climbing assay of *Oregon-R* flies shows a reduced climbing ability in the rotenone-fed group compared to control in (A). Two-way ANOVA, followed by a Tukey’s multiple comparison test, was used to analyse statistical significance in climbing abilities between control and rotenone-fed groups with P-values: ns (not significant), *0.0332, **0.0021, and ****<0.0001 (A). The climbing assay of *Canton-S* flies shows an increased climbing ability in the rotenone-fed group compared to control in (B). Two-way ANOVA, followed by a Tukey’s multiple comparison test, was used to analyse statistical significance in climbing abilities between control and rotenone-fed groups with P-values: ns (not significant), **0.0021, and ****<0.0001 (B). Lifespan assay of *Oregon-R* and *Canton-S* flies when fed with rotenone-supplemented food (C). Rot, OR, and CS are abbreviations for rotenone, Oregon-R, and Canton-S.

## Description

PD is the second most common neurodegenerative disorder worldwide, with no definite cure. PD is characterized by the selective loss of the dopaminergic neurons in the substantia nigra pars compacta (SNpc) region of the brain. This selective loss of the dopaminergic neurons in the SNpc is attributed to the formation of Lewy bodies in the brain and in various other regions in the patients’ body (Rizek et al., 2016) . The Lewy bodies’ presence is the hallmark of PD, and is mainly constituted by a protein called, α-synuclein, which if gets misfolded, starts to clump into fibrils, eventually forming Lewy bodies (Rizek et al., 2016) . The symptoms of PD are both motor and non-motor; motor symptoms include bradykinesia, rigidity, resting tremor, and postural instability while non-motor symptoms include cognitive decline, gut dysbiosis, depression, anxiety, and sleep disorders (Kalia and Lang 2015; Postuma et al., 2015) .

Many studies have reported that PD's onset, progression, and symptoms differ between individuals residing in different geographical regions, specifically Eastern and Western countries (Abbas et al. 2018). On a shorter scale, there are reports of differences in the prevalence of PD limited within the North American continent and even within the US itself (Marras et. al, 2018). These differences in the prevalence of the disease can be due to misdiagnosis, no diagnosis at all in many cases, or purely due to geographical differences (Marras et. al, 2018).

Statistically, more than 95% of PD cases are sporadic where the cause of disease onset is not known. In such cases, it is thought that the onset is due to exposure to environmental pollutants, chemicals, toxins such as MPTP, paraquat, or rotenone (Van Laar et al., 2023) . Although exposure to such toxins is involved in the onset of sporadic PD, growing evidence suggests that the interplay between genetic and environmental factors together are responsible for the onset of the disease (Tran et al., 2020) . However, it remains unclear whether these genetic factors are mutations or predispositions (Tran et al., 2020) . Thus, the understanding of the mechanism(s) underlying the intricate interplay between genetic susceptibilities and environmental factors are important for improved treatments and diagnostic strategies against the disease (Tran et al., 2020) .

We have generated a sporadic PD model by rotenone-supplemented feeding in *Drosophila melanogaster* to mimic the symptoms of PD. Rotenone is a naturally occurring lipophilic, plant-based compound that is derived from the roots of *Derris spp.* , *Lonchocarpus spp.,* and *Tephrosia spp* . Even though its use in herbicides and pesticides has been banned in the US, Europe, and Canada, it is still reportedly in use in many countries (Heinz et al. 2017; Zhang et al. 2022; Miller et al., 2023) . Chronic exposure of rotenone in rodent and fly models has been shown to reproduce the symptoms of PD including selective loss of dopaminergic neurons, formation of similar protein aggregates as Lewy bodies, and locomotory defects (Miller et al., 2023; Shere et al. 2003) . Rotenone causes PD by inhibiting the complex I of the mitochondrial electron transport chain (ETC) causing electron leakage, and subsequently impairing the oxidative phosphorylation (OXPHOS) system. The OXPHOS system impairment generates reactive oxygen species (ROS), and these ROS ultimately cause the degeneration of neurons in the SNpc by lipid peroxidation, direct DNA damage (Feany and Bender 2000; Roy et al., 2023).

The present study reports differential responses of two wild-type *Drosophila melanogaster* strains, *Oregon-R and Canton-S* with different geographical origins, to chronic rotenone exposure. Here, we evaluate the motor defects and viability of the two wild-type *Drosophila melanogaster* strains upon chronic exposure to rotenone in an age-dependent manner (40 days). After 40 days of being fed with rotenone, it was observed that the *Oregon-R* flies exhibited a significant decline in climbing ability compared to the age-matched *Canton-S* flies (Fig 1A and 1B). Conversely, over 40 days, *Canton-S* flies had a lower survival rate than the age-matched *Oregon-R* flies (Fig 1C). These results are consistent with the reports discussing manifestations of PD in individuals where environmental impacts are influenced by genetic predisposition(s) along with the involvement of epigenetic changes occurring in the genome (Tsalenchuk et al., 2023; Pang et al., 2019).

Clarifying the genetic components causing these variations and their similarities to the pathophysiology of PD in humans is yet to be investigated. To further understand the molecular basis of this differential response to rotenone, genomic and transcriptomic studies can be done in different strains of *Drosophila* to enhance the understanding of PD in humans.

## Methods

*Fly stocks and Maintenance*

Wild-type *Oregon-R* (BL – 25211) and *Canton-S* (BL – 64349) strains were used throughout the study. All flies were kept on a cornmeal-agar-sugar medium at 25 °C with a 12-hour light/dark cycle.

*Media Preparation and Rotenone Treatment*

The cornmeal-agar-sugar media was prepared by mixing corn flour (94.7 gm), sugar (83.3 gm), yeast (33.3 gm), and agar powder (Himedia, Cat-no GRM026-500G; 13.8 gm) in 2 L of water and subsequent boiling of the mixture. After boiling the mix, it was allowed to become lukewarm. Propionic acid (SRL, Cat-no 42263; 5.4 mL in 2 L) and methyl-4-hydroxybenzoate (SRL, Cat-no 44055; 5.4 gm in 2 L) were added to the lukewarm media as anti-fungal and anti-bacterial agents, respectively, and the media was subsequently stirred thoroughly. About 4–5 mL of the prepared media was poured into smaller food vials which were used to culture the flies. The corn flour, sugar, and yeast used in the media was purchased by local vendors. Rotenone (Sigma-Aldrich, Cat-no. R8875) was directly mixed into the cornmeal-agar-sugar medium to a final concentration of 500 µM after the addition of propionic acid and methyl-4-hydroxybenzoate and stirred constantly while pouring into the vials to ensure proper distribution of rotenone in each vial. Thirty-two flies (three to four days old) per vial were kept in a male-to-female ratio of 1:3 in both control and rotenone-fed groups. Control groups were fed normal (rotenone-free) cornmeal-agar-sugar medium.

*Behavioural Assays and Lifespan Assays*

The climbing assay was performed to determine the locomotor activity of the flies (Narwal et al. 2024) . This was carried out by anesthetizing the flies and randomly selecting 10 flies from each biological replicate of 32 flies maintained in each vial and placing them in a vertical column or test tube. The selected flies were left in the vertical column or test tube for about 10–15 minutes at room temperature for revival and acclimatization (Narwal et al. 2024) . The flies were gently tapped to the bottom of the column, and the number of flies climbing up to and beyond the 8 cm mark within 10 seconds was recorded. Climbing assays were performed at seven different time points of 3, 7, 14, 21, 30, and 40 days (Fig 1A and 1B). At each time point, the climbing assay was performed at least three times in a row using the same set of 10 flies from each biological replicate. The percentage of the number of flies were calculated and the mean of the percentage values were used as the overall value for each single biological replicate. Further the values were normalized to that of control and values were obtained in terms of fold change. This method was consistent for all three biological replicates at each time point.

Similarly, three biological replicates of 32 flies per group were used in the lifespan assay. The daily count of surviving flies was determined, and the data were used to plot the survival curves (Fig 1C).

*Statistical Analyses*

GraphPad Prism 8.0.1 was utilized for statistical analysis and graphical representation of the data, and the significance was expressed as P values, determined through two-way ANOVA, followed by Tukey's multiple comparison tests (Narwal et al. 2024) .

Each result is representative of at least three biological and three technical repeats.
